# Mechanistic mathematical modelling of mercaptopurine effects on cell cycle of human acute lymphoblastic leukaemia cells

**DOI:** 10.1038/sj.bjc.6602893

**Published:** 2005-12-06

**Authors:** J C Panetta, W E Evans, M H Cheok

**Affiliations:** 1 Department of Pharmaceutical Sciences, St. Jude Children's Research Hospital, 332 North Lauderdale St., Memphis, TN 38105, USA; 2 University of Tennessee, Memphis, TN, USA

**Keywords:** cell cycle, mercaptopurine, mathematical model

## Abstract

The antimetabolite mercaptopurine (MP) is widely used to treat childhood acute lymphoblastic leukaemia (ALL). To study the dynamics of MP on the cell cycle, we incubated human T-cell leukaemia cell lines (Molt-4 sensitive and resistant subline and P12 resistant) with 10 *μ*M MP and measured total cell count, cell cycle distribution, percent viable, percent apoptotic, and percent dead cells serially over 72 h. We developed a mathematical model of the cell cycle dynamics after treatment with MP and used it to show that the Molt-4 sensitive controls had a significantly higher rate of cells entering apoptosis (2.7-fold, *P*<0.00001) relative to the resistant cell lines. Additionally, when treated with MP, the sensitive cell line showed a significant increase in the rate at which cells enter apoptosis compared to its controls (2.4-fold, *P*<0.00001). Of note, the resistant cell lines had a higher rate of antimetabolite incorporation into the DNA of viable cells (>1.4-fold, *P*<0.01). Lastly, in contrast to the other cell lines, the Molt-4 resistant subline continued to cycle, though at a rate slower relative to its control, rather than proceed to apoptosis. This led to a larger S-phase block in the Molt-4 resistant cell line, but not a higher rate of cell death. Gene expression of apoptosis, cell cycle, and repair genes were consistent with mechanistic dynamics described by the model. In summary, the mathematical model provides a quantitative assessment to compare the cell cycle effects of MP in cells with varying degrees of MP resistance.

The thiopurine antimetabolites mercaptopurine (MP) and thioguanine are analogues of the purine bases hypoxanthine and guanine and these medications are a major component of paediatric acute lymphoblastic leukaemia (ALL) treatment protocols worldwide. After cellular uptake, the first essential activation step is catalysed by hypoxanthine-guanine phosphoribosyl transferase (HPRT). Phosphoribosylation activates MP through the purine ‘salvage pathway’ via TIMP (6-thioinosine-5′-monophosphate), and TXMP (6-thioxanthosine-5′-monophosphate) to thioguanine nucleotides (TGNs) which are considered to be the main active metabolites (Relling *et al*, 1999). Thiopurine-S-methyltransferase (TPMT) catalyses S-methylation of MP and MP-metabolites, that is the main intracellular inactivating pathway in haematopoietic tissues. However, the most abundant S-methylated MP metabolite, S-methylthioinosine-5′-monophosphate (^me^TIMP), inhibits phosphoribosyl pyrophosphate (PRPP)-amidotransferase, an essential enzyme in the *de novo* purine synthesis pathway (DNPS). The MP pathway is given in Figure 1A, illustrating the above-described enzymes.

Thiopurine cytotoxicity is explained in part by inhibition of DNPS, which is essential for generating new purines for DNA and RNA synthesis. However, incorporation of TGNs into DNA and RNA is generally considered to be the principal mechanism of MP cytotoxicity. Primarily, 2′-deoxy-6-thioguanosine-5′triphosphate (dG^S^TP) is incorporated into DNA, subsequently showing a characteristic delayed cytotoxic effect (Maybaum and Mandel, 1983). Recent studies have shown that the incorporation of dG^s^TP into duplex DNA results in subtle localised modification on DNA structure (Somerville *et al*, 2003). Moreover, chromatin structure alteration is most evident in the G2 phase of the cell cycle (Maybaum and Mandel, 1983). Enzymes necessary for DNA replication are affected by MP incorporation into DNA, including DNA polymerase, DNA ligase I (Ling *et al*, 1992), topoisomerase II (Krynetskaia *et al*, 2000) and RNase H (Krynetskaia *et al*, 1999). Furthermore, post-replicative mismatch repair (MMR) proteins may play a role in MP mechanism of action (Waters and Swann, 1997). Mismatch repair proteins can ‘tag’ mismatches, the dG^S^TP^*^T pair among them, and futile attempts to repair this DNA mismatch is thought to trigger apoptosis. More recently, a novel protein complex containing HMGB1, HMG2, HSP70, ERp60 and GAPDH has been shown to bind preferentially to duplex DNA into which a dG^S^TP has been incorporated (Krynetski *et al*, 2003). However, the precise molecular mechanism(s) of MP cytotoxicity and resistance is not completely understood.

Acquired or intrinsic drug resistance mechanisms extensively studied in anticancer treatment are enhanced efflux through activation of transmembrane proteins, increased detoxification (e.g., via activation of glutathione transferases) and activation of anti-apoptosis or cell cycle arrest through survival signals such as Bcl-2 or p53. These specific examples have not been identified as resistance mechanisms to MP in leukaemia, although in principle they may apply. Previously studied resistance mechanisms are ABCB1 (MDR1) (Zeng *et al*, 2004), ABCC4 and ABCC5 (MRP4 and MRP5) (Reid *et al*, 2003) and MMR deficiency (Offman *et al*, 2004).

Given cell cycle kinetic information including cell cycle phase distribution and percentage of cells in apoptosis, mathematical models can be used to test hypotheses on the mechanism of action of the drug and to point to possible drug-resistance mechanisms. Mathematical models have been previously used to understand dose dependency of cell cycle dynamics and to quantitate differences in cell cycle block and apoptosis of an experimental anti-mitotic agent (Kozusko *et al*, 2001). The objective of the current study is to investigate, via mathematical models and gene expression analysis, the effects of MP on the cell cycle dynamics of three ALL cell lines that differ in their sensitivity to MP.

## MATERIALS AND METHODS

### Cell lines

Two human T-cell leukaemia cell lines were used, that are commercially available, representing MP sensitivity: Molt-4 (American Type Culture Collection, Rockville, MD, USA) and MP resistance: P12 (German Collection of Microorganism and Cell Cultures, Braunschweig, Germany) and a subline of Molt-4 that was not intentionally selected for MP resistance. The media consists of 89% RPMI 1640, 1% L-Glutamine, 200 mM (BioWhittaker, Walkersville, MD, USA), and 10% fetal bovine serum (HyClone, Logan, UT, USA).

### Mercaptopurine treatment

In each experiment, MP (Sigma, St Louis, MO) stock solution was freshly prepared. Cells of 0.5 to 1 × 10^6^ cells ml^−1^ density were incubated in 10 *μ*M final concentration of MP for 12, 24, 48, and 72 hours at 37°C in 5% CO_2_. The IC_50_s (concentration that inhibits 50% of cell growth relative to controls) for the three cell lines for the 24-h MP exposure was determined by flow cytometric analysis.

### Flow cytometric analyses

To measure total cell count, cell cycle distribution, percent viable, percent apoptotic, and percent dead cells, cells were stained with annexin-V and propidium iodide (PI) and analysed by flow cytometry (FACS) with the Coulter EPICS-V flow cytometer (Coulter Electronics Inc., Hialeah, FL, USA). The computer program PEAK was used to calculate the percentages of cells in the G0/G1, S, G2/M phase. Controls without MP were also run for each cell line at the same sampling times.

### Mathematical modelling

The model developed here is a modification of a previous model of cell cycle dynamics used to describe a M-phase specific anticancer agent (Kozusko *et al*, 2001). These modifications account for the unique dynamics of MP and the specific form of the experimental results available in this study, that is, total cell count, percent G0/G1-phase, percent S-phase, percent G2/M-phase, percent viable, percent apoptotic, and percent non-viable. Figure 2 shows the dynamics of the model, which is described by the following system of ordinary differential equations: 
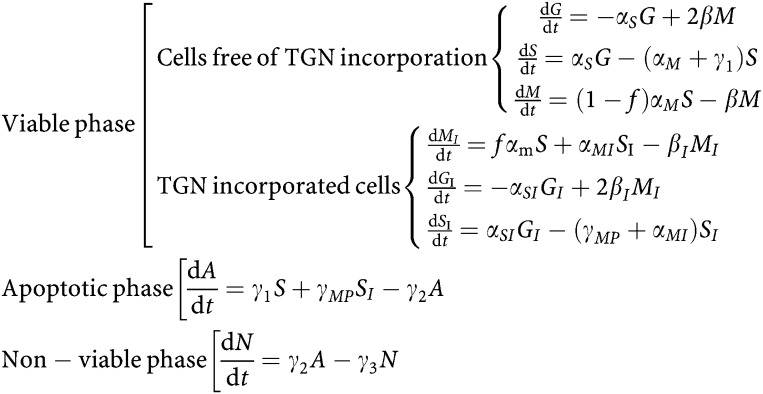


The variables are as follows: *G*, number of cells in G0/G1-phase; *S*, number of cells in S-phase; *M*, number of cells in G2/M-phase; *G*_*I*_, *S*_*I*_, and *M*_*I*_ are the equivalent variables for the TGN incorporated phases; *A*, apoptotic cells; *N*, non-viable cells. All the parameters describe the rate of transition (1/hour) between phases. 
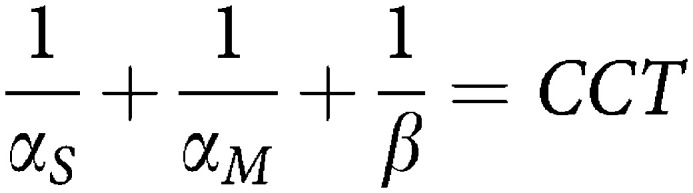


*CCT* is the cell cycle time (defined in terms of transition rate parameters in the above equation) in hours; *α*_*S*_, *α*_*SI*_, *β*, *β*_*I*_, *α*_*M*_, and *α*_*MI*_ all describe transition rates between cell cycle phases; *f*∈[0,1] describes the fraction of cells that continue through the cell cycle at least one more cycle after TGNs were incorporated into their DNA before going through apoptosis (its magnitude is an indication of S-phase block efficiency); *γ*_1_ and *γ*_*MP*_ describe the transition rate of *S* and *S*_I_-phase cells to apoptosis, respectively; *γ*_2_ describes the transition rate of apoptotic cells to the non-viable state; and *γ*_3_ describes the transition rate out of the non-viable state.

### Parameter estimation

To estimate the parameters, the model was first fit to the control data for each of the cell lines to obtain estimates for the model parameters *α*_*S*_, *β*, *α*_*M*_, *γ*_1_, *γ*_2_, and *γ*_3_. In all cases, *CCT* was fixed (which in turn set *β*) to allow a unique solution for the remaining parameters. Once these parameters were obtained, *α*_S_, *β*, and *α*_M_ were fixed and the remaining parameters were estimated using the MP-treated data. In all cases, the maximum likelihood parameter estimation method as implemented in ADAPT II (D’Argenio and Schumitzky, 1997) was used. Parameter estimation accuracy was given in terms of the coefficient of variation (CV in percent). Comparisons between control and treated parameters and between cell lines were made using the *t*-test.

### Gene expression profiling

Molt-4 sensitive and Molt-4 resistant cells were also subjected for gene expression analysis. RNA of 5 × 10^6^ cells was isolated by phenol/guanidine thiocyanate extraction using TRI REAGENT (Molecular Research Center, Cincinnati, OH, USA). High-quality cRNA was hybridised to the HG-U95Av2 oligonucleotide microarray, which contains 12 599 gene probe sets, representing approximately 9670 known genes, plus approximately 1495 EST clones (Affymetrix, Santa Clara, CA, USA). Scaled gene expression values for pretreatment, post-treatment (10 *μ*M MP for 24 hours) and fold-change (post-treatment *vs* pretreatment ratio) were calculated using the default settings of Affymetrix Microarray Suite software version 5 (MAS 5.0) (Cheok *et al*, 2003). Pathway genes were selected using Netaffx. Pathway analysis was performed using Ingenuity software (http://www.ingenuity.com). The probe sets were mapped to the corresponding gene within the Ingenuity Pathway Knowledge Base.

## RESULTS

**Figure 1:**
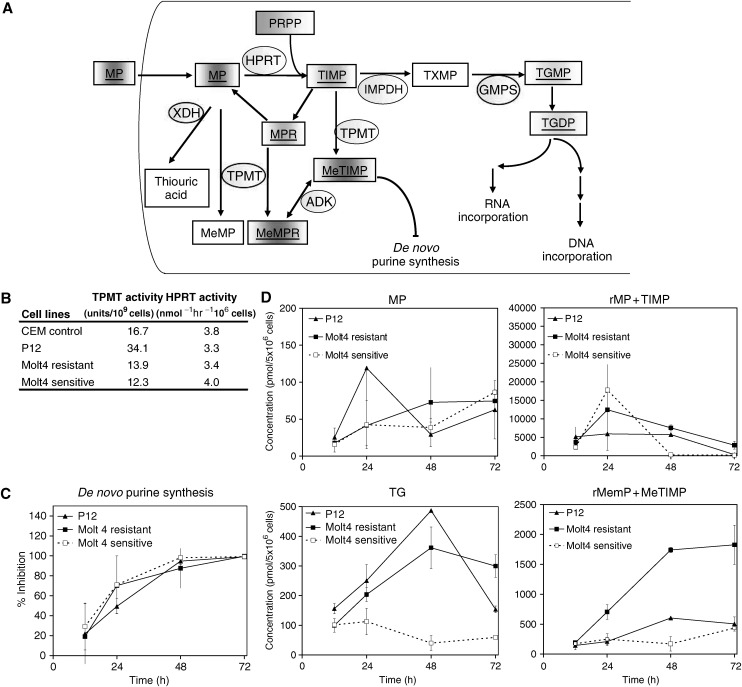
(**A**) Mercaptopurine metabolic pathway. Intracellular mercaptopurine (MP) is converted into thioinosine monophosphate (TIMP) by hypoxanthine-guanine phosphoribosyl transferase (HPRT), using 5-phospho-D-ribose-1-pyrophosphate (PRPP). TIMP is converted into thioxanthosine monophosphate (TXMP), then to thioguanosine monophosphate (TGMP), by inosine monophosphate dehydrogenase (IMPDH) and guanine monophosphate synthetase (GMPS). TGMP can be converted into thioguanine nucleotide diphosphate (TGDP) and triphosphate (TGTP). Cytotoxic effects occur when TGN is incorporated into DNA or RNA or when methylthioinosine monophosphate (MeTIMP) inhibits *de novo* purine synthesis. The inactivation of MP is catalysed by xanthine oxidase (XDH), thiopurine methyltransferase (TPMT) or adenosine kinase (ADK). Alternative products are mercaptopurine riboside (rMP), methylmercaptopurine (MeMP), methylmercaptopurine riboside (rMeMP). Enzymes are shown in circles and metabolites in boxes, underlined are metabolites detected in our assay. (**B**) TPMT and HPRT activity are listed for the three cell lines. (**C**) DNPS inhibition following MP treatment was measured in three cell lines at the sampling hours indicated. (**D**) Shows intracellular MP metabolite concentration in the three cell lines P12 resistant, Molt-4 resistant, and Molt-4 sensitive at the sampling hours indicated. Measured were MP, rMP+TIMP, all thioguanine nucleotides (TG), rMeMP+MeTIMP.

To compare the relative resistance among the three leukaemia cell lines we determined the IC_50_s after 24-hours incubation with MP: Molt-4 sensitive, 6–10 *μ*M; P12, >50 *μ*M; and Molt-4 resistant, >50 *μ*M. Initial TPMT and HPRT activity were measured and no difference was found for HPRT activity, for P12 cells TPMT activity was higher compared with the other lines (Figure 1B) (Dervieux *et al*, 2001). DNPS inhibition after MP exposure is a mechanism that may contribute to cytotoxicity, but was not found different in these three cell lines (Figure 1C) (Dervieux *et al*, 2002). Additionally, intracellular metabolites of MP were measured (Dervieux *et al*, 2002) (Figure 1D) and drug resistance cannot be attributed to reduced uptake of MP (Figure 1D, top panels) or lower level of active metabolites (TGN), as the sensitive Molt-4 in fact accumulates lower level of TGN compared to the resistance cell lines (Figure 1D, Panel TG). Molt-4 resistant cells accumulate higher level of methylated metabolites (Figure 1B, Panel rMeMP, MeTIMP) in contrast to the other resistant cell line P12 or to the sensitive Molt-4. Therefore, we anticipate differences in cell cycle dynamics related to mechanisms other than DNPS or MP metabolism in these three lines.

**Figure 2:**
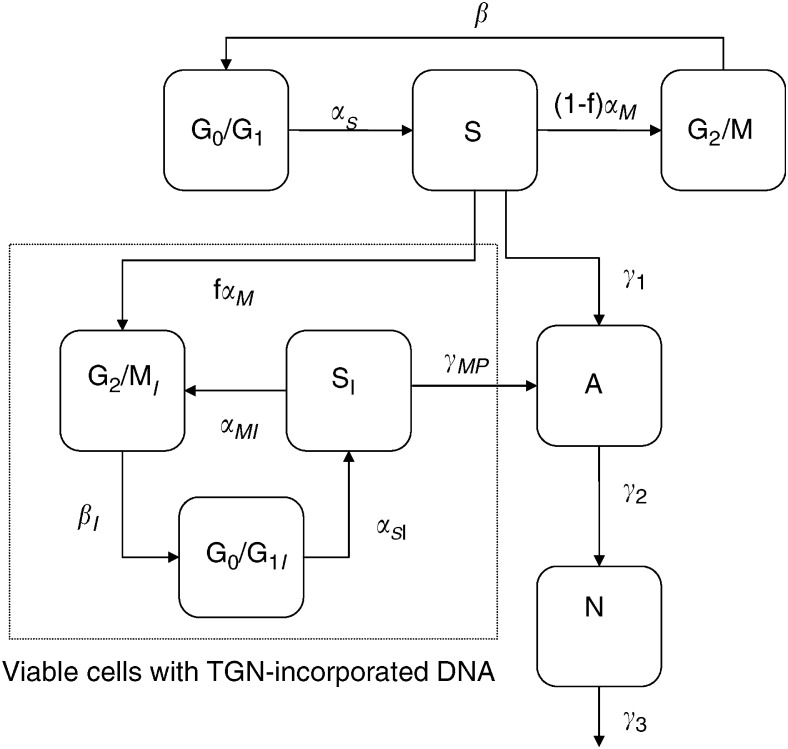
Schematic of mathematical model describing the dynamics of MP. The variables and parameters are defined in the text.

**Figure 3:**
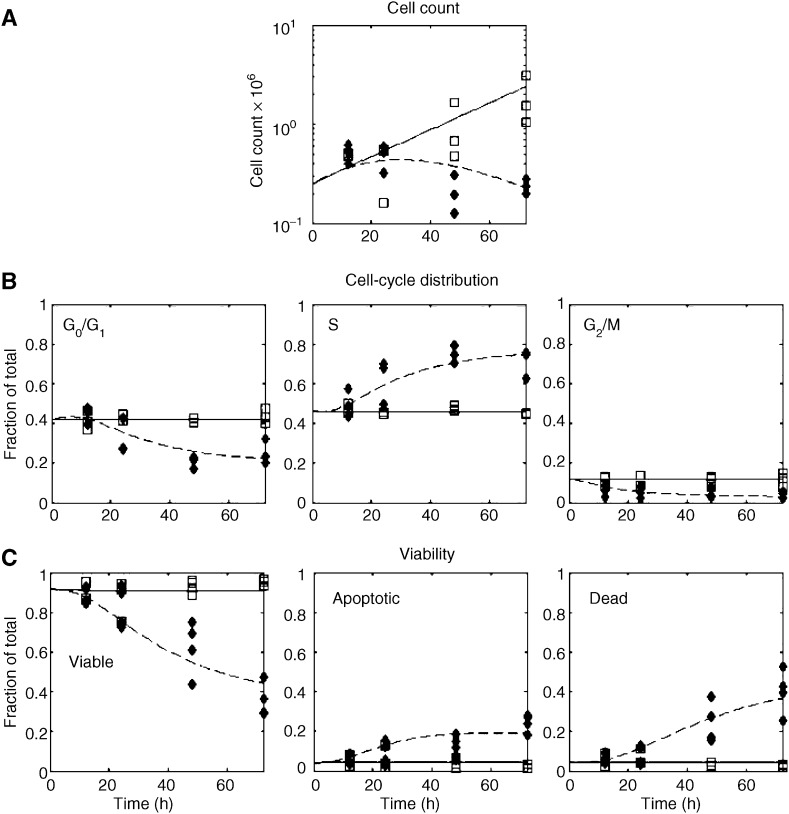
P12 cell line: -□- Control data. -⧫- MP-treated data. The solid line represents the model fit to the control data and the dashed line represents the model fit to the MP-treated data. (**A**) Total cell count *vs* time in hours. (**B**) Cell cycle distribution (i.e., fraction G0/G1, S, G2/M, respectively). (**C**) Cell viablility distribution (i.e., fraction viable, fraction apoptotic, and fraction dead, respectively).

**Figure 4:**
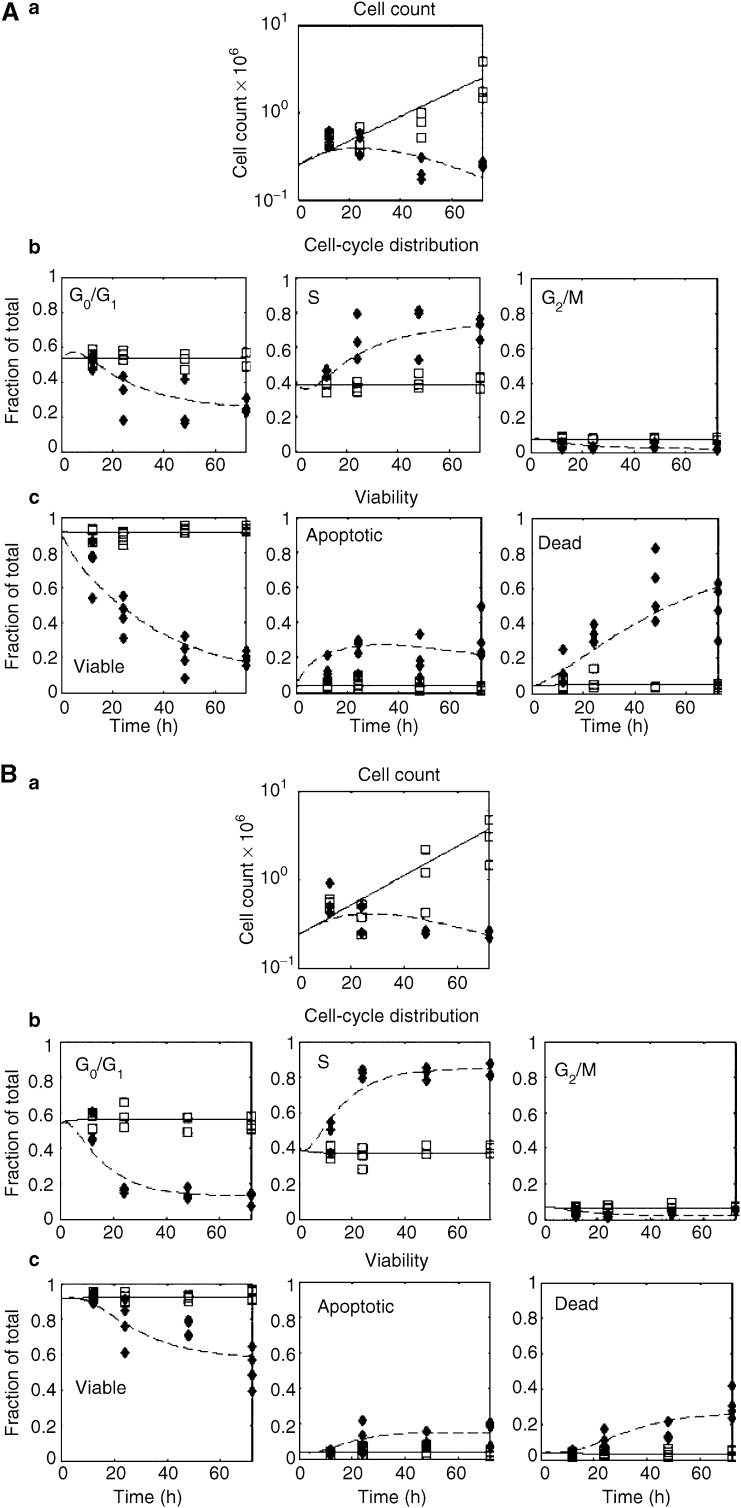
Molt-4 cell line. (**A**) Molt-4 sensitive cell line and (**B**) Molt-4 resistant cell line: -□- Control data. -⧫- MP treated data. The solid line represents the model fit to the control data and the dashed line represents the model fit to the MP-treated data. Panel a: Total cell count *vs* time in hours. Panel b: Cell cycle distribution (fraction G0/G1, S, G2/M, respectively). Panel c: Cell viability distribution (i.e., fraction viable, fraction apoptotic, and fraction dead, respectively.

Using the model described in Figure 2, each of the three cell lines we considered showed distinct dynamics. These are illustrated in Figures 3 and 4, where the experimental data is shown along with the model fit. The parameter estimates for all the fits are given in Table 1. Models other than the one described were also considered, but they were inadequate in describing the data. For example, one model considered fixing *f*=*0* which means that once TGNs are incorporated into the cells, cells immediately enter apoptosis. However, with this assumption, the model did not describe the block in S-phase appropriately and failed in particular to describe the 12–24 hour delay in the initiation of the cell cycle block (data not shown).

To better understand the different dynamics between these cell lines, we focused on the parameters that either exhibit large changes between the control and MP-treated states or exhibit large changes among cell lines. There were three main parameters (or groups of parameters) where we observed significant differences. These included the parameters representing apoptosis (*γ*_1_, *γ*_2_, *γ*_3_, and *γ*_*MP*_), the parameter representing TGN incorporation (*f)*, and lastly, the parameters representing the viable cell dynamics after TGN incorporation (*α*_*SI*_, *β*_*I*_, and *α*_*MI*_). Differences in these model parameters are indicative of the different mechanisms of action of MP in the cell lines studied.

When considering the untreated state for each cell line, the MP-sensitive cell line (Molt-4 sensitive) had a significantly higher rate of cells entering apoptosis (*γ*_1_, 2.7 and 3.3-fold, *P*<0.00001), transiting through apoptosis to the non-viable phase (*γ*_2_, 2.9 and 4.3-fold, *P*<0.00001), and eliminating from the non-viable phase (*γ*_3_, 2.8 and 4.0-fold, *P*<0.00001) relative to the resistant cell lines (P12 and Molt-4 resistant). When treated, the sensitive cell line (Molt4 sensitive) showed a significant increase, compared to the control, in the rate at which cells immediately enter apoptosis after TGN incorporation (*γ*_1_, increased 2.4-fold, *P*<0.00001). In addition, the rate of apoptosis in viable cells with incorporated TGN was highest in Molt-4 sensitive, intermediate in P12 and lowest in Molt-4 resistant cells (*γ*_MP_ for Molt-4 sensitive 1.7 and 1.9-fold higher, *P*<0.00001, relative to P12 and Molt-4 resistant cells).

When treated with MP, the model parameters indicated that the more resistant cell lines (P12 and Molt-4 resistant) had a higher rate of TGN incorporation into the DNA of viable cells (*f*, increased 1.4 and 2-fold, *P*<0.01) relative to the Molt-4 sensitive cell line. This is in line with the experimental results on TGN incorporation in Figure 1D, Panel TG. Both P12 and Molt-4 sensitive cell lines completed only one additional cell cycle after MP exposure (with a time to complete the additional cycle of 9.4 and 7.8 hours, respectively) before proceeding to apoptosis (*α*_*MI*_=0) and showed similar S-phase blocks (related to no significant difference in *α*_SI_). In contrast, the Molt-4 resistant subline continued to cycle (*α*_*MI*_>0, *P*<0.00001), though at a rate eight times slower relative to the controls (*α*_*M*_/*α*_*MI*_=8, *P*<0.00001). This led to a cell cycle time (CCT) in the TGN-incorporated cells that were 3.9 times slower then their controls. This, along with the above-mentioned lower rate of apoptosis in viable cells with TGN incorporated, led to a larger S-phase block in the Molt-4 resistant cell line, but not a higher rate of cell death.

**Figure 5:**
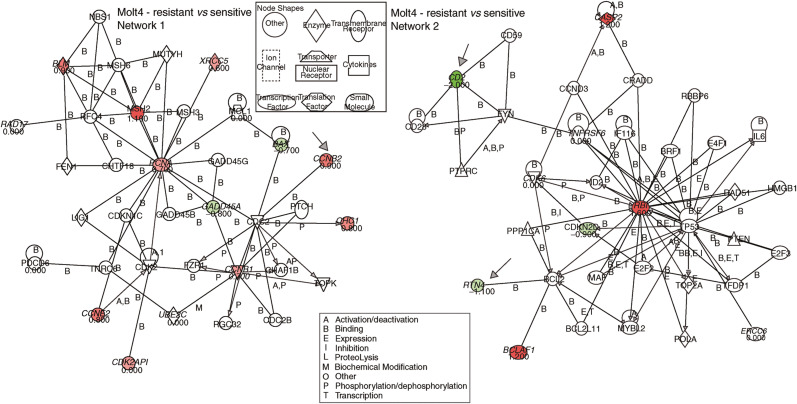
Pathway analysis of genes that were differentially expressed in resistant compared to sensitive Molt-4 ALL cells. Arrows indicate genes discussed in the text. Forty-three probe sets (28 unique genes) were differentially expressed between resistant and sensitive Molt-4 ALL cells or changed expression before and after treatment with MP in resistant compared to sensitive Molt-4 ALL cells. These genes served as the input for the pathway query. Two networks were found that included the input genes, labelled in green if expression is lower in resistant compared to sensitive Molt-4 ALL cells and red if expression is higher, respectively. The number below the gene indicates the fold-change.

**Figure 6:**
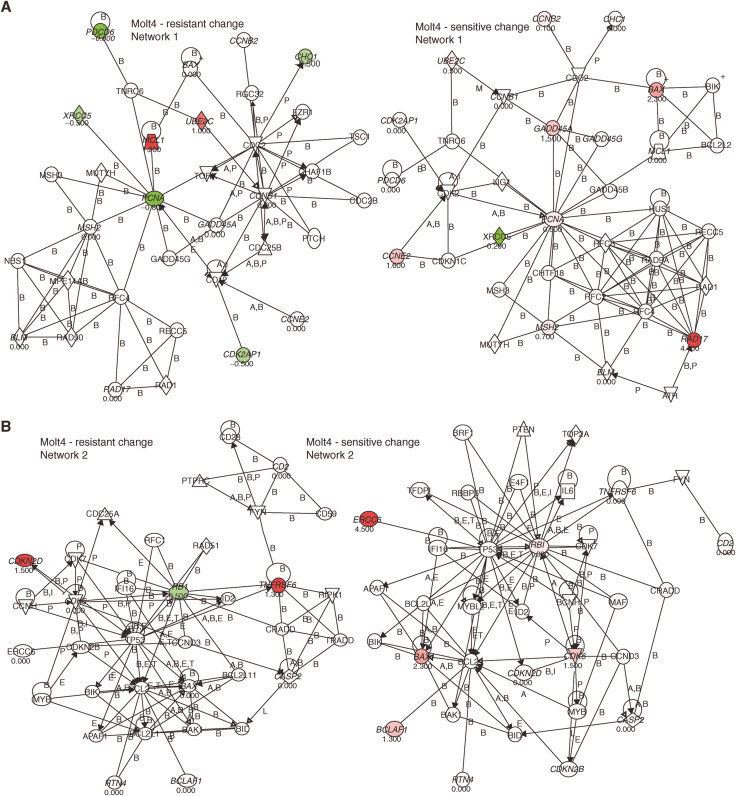
Pathway analysis of genes that changed expression before and after treatment with MP in resistant and in sensitive Molt-4 ALL cells. Arrows indicate genes discussed in the text. Forty-three probe sets (28 unique genes) were differentially expressed between resistant and sensitive Molt-4 ALL cells or changed expression before and after treatment with MP in resistant compared to sensitive Molt-4 ALL cells. These genes served as the input for the pathway query. Two networks were found that included the input genes (Network 1 shown in (**A**) and Network 2 in (**B**), respectively), genes labelled in green if expression decreased after MP treatment and red if expression increased after MP treatment and the number below the gene indicates the fold-change.

Gene expression analysis pre- and post-treatment was performed on Molt-4 resistant and Molt-4 sensitive cells. We extracted all gene probe sets from the U95Av2 microarray that were involved in the purine pathway (*n*=103), cell cycle regulation (*n*=561), apoptosis (*n*=407) or repair (*n*=192), based on the NetAffx™ analysis center database (http://www.affymetrix.com). Of these 241 were duplicates, 24 were not detected in all four chips (absent by Affymetrix detection call), and 763 did not show a significant change in all three comparisons (no change by Affymetrix change call). Figures 5 and 6 summarise the results indicating the genes with relative changes (>50%) checked by absolute changes (signal >500) along with a graphical description of the pathway analysis. In summary, pro-apoptotic genes were under-expressed in Molt-4 resistant compared to Molt-4 sensitive (e.g., *BAX*, *RTN4, CD2*), upregulated after MP treatment in Molt-4 sensitive (e.g., *BAX*, *BCLAF1*) and downregulated after MP treatment in Molt-4 resistant cells (e.g., *PDCD2, PDCD6*). In contrast, anti-apoptotic genes were overexpressed in resistant cells compared to sensitive cells (*CASP2*) and upregulated in MP-resistant cells after treatment with MP (e.g., *MCL1, TNFRSF6*). Of the genes involved in cell cycle regulation cyclin B1, B2, E2 and RB 1 were overexpressed in resistant cells and cyclin E2 and *CDK6* were induced in sensitive cells after treatment with MP. Lastly, repair genes were overexpressed in resistant cells compared to sensitive cells (e.g., *MSH2, BLM*), downregulated after treatment (e.g., *PCNA, XRCC5*) or upregulated (*PMS2L3, 5, 6*) in resistant cells or upregulated after treatment in sensitive cells (e.g., *GADD45A, ERCC6, RAD17, MSH2*).

## DISCUSSION

The mathematical model of the cell cycle dynamics of MP described in this study is useful to test hypotheses related to potential causes of drug resistance to MP. In particular, it illuminated differences in resistance to MP based on altered growth dynamics among cell lines during treatment with MP. The model is particularly useful in understanding dynamics, which are not intuitive from the experimental data. Our model focuses on MP resistance owing to alterations in cell cycle regulation.

One finding of our model is that the P12 cell line and the resistant Molt-4 subline arrest efficiently in S-phase after incorporating MP but have a lower rate of cells entering apoptosis relative to the MP-sensitive Molt-4 cell line. Furthermore, the resistant Molt-4 subline has the ability to continue cycling after incorporation of TGN without going into apoptosis. This suggests that in this resistant cell line, the mechanism of resistance is not a lack of TGNs being incorporated into the DNA, but rather a faulty mechanism which does not detect incorporated TGNs and/or faulty mechanism of signaling apoptosis. These dynamics point to a possible defect in detecting the DNA/RNA damage. Thus, extended S-phase arrest is possibly an indication of MP resistance.

These observed differences in apoptosis may be owing to alterations in the MMR system (e.g., MSH2 (Krynetskaia *et al*, 2003)) Sensitive cell lines may have higher expression of these proteins or may induce expression after treatment more efficiently; they may be absent in resistant cell lines or may reduce expression after treatment. In fact, expression array data show a larger increase of MSH2 expression in the Molt-4 sensitive cell line compared to the resistant subline (after treatment relative to controls, Figure 6). Thus, if the MMR system is not functioning appropriately in the resistant cell lines then, as the model predicted and as gene expression confirmed, TGNs may be incorporated into the DNA but because these cells lack mismatch detection it continues to remain viable as opposed to entering apoptosis as the sensitive cells do.

Additionally, decreased pro-apoptotic signalling in Molt-4 resistant compared to Molt-4 sensitive (e.g., *BAX*, *RTN4, CD2*), downregulation after MP treatment in Molt-4 resistant cells (e.g., *PDCD2, PDCD6*) and upregulation after MP treatment in Molt-4 sensitive (e.g., *BAX, BCLAF1*) point to decreased apoptosis in the Molt-4 resistant increased apoptosis in the Molt-4 sensitive cells pre- and post-treatment, as presented by the model. Consistent with these findings was the expression of genes involved in anti-apoptotic signalling as they were overexpressed in resistant cells compared to sensitive cells (*CASP2*) and upregulated in MP-resistant cells after treatment with MP (e.g., *MCL1, TNFRSF6*).

Lastly, continuation of cycling of Molt-4-resistant cells may be due to cyclin B1, B2, E2 and RB 1 that were overexpressed in resistant cells and downregulation of PCNA after MP treatment compared to sensitive cells.

The results of our modelling study point to key regulators in the cell cycle signalling pathway that pharmacodynamically affect sensitivity and/or resistance of ALL cells to MP.

## Figures and Tables

**Table 1:** Model parameter estimates

	**P12**		**Molt-4 sensitive**		**Molt-4 resistant**	
**Parameter**	**Control**	**Treated**	**Control**	**Treated**	**Control**	**Treated**
*α*_*S*_	0.1559 (4.8)^a^		0.1588 (4.0)^a^		0.1182 (3.8)^a^	
*α*_*M*_	0.0941 (3.3)^a^		0.1388 (3.7)^a^		0.1238 (4.1)^a^	
CCT^b^	20.0		15.0		18.0	70.8
						
*γ*_1_	0.0184 (11.6)^a^	NSC*	0.0503 (5.5)^a^	0.1210 (10.0)^a^	0.0152 (15.6)^a^	NSC*
*γ*_2_	0.1438 (18.2)^a^	NSC*	0.4118 (11.8)	0.0829 (6.1)	0.0956 (25.5)^a^	0.1555 (9.2)^a^
*γ*_3_	0.1064 (23.3)^a^	NSC*	0.2915 (11.3)	0.0485 (8.7)^a^	0.07329 (37.3)^a^	0.1045 (10.7)^a^
						
*f*		0.7277 (3.4)^a^		0.5080 (7.7)^a^		1.0
*α*_*SI*_		NSC*		NSC*		0.2289 (10.1)^a^
*α*_*MI*_		0		0		0.0154 (15.3)^a^
*γ*_*MP*_		0.0671 (6.9)^a^		0.0758 (9.0)^a^		0.0402 (7.5)^a^

a Coefficient of variation (%).

b Cell cycle time (h).

* No significant change.
